# Neurodevelopmental profiles of preschool-age children in Flint, Michigan: a latent profile analysis

**DOI:** 10.1186/s11689-021-09377-y

**Published:** 2021-08-19

**Authors:** Shuting Zheng, Somer L. Bishop, Tiffany Ceja, Mona Hanna-Attisha, Kaja LeWinn

**Affiliations:** 1 Department of Psychiatry and Behavioral Sciences, Weill Institute for Neurosciences, University of California San Francisco, 401 Parnassus Ave, San Francisco, CA 94143 USA; 2 Division of Public Health, Pediatric Public Health Initiative, Michigan State University, Flint, MI 48502 USA; 3 Department of Pediatrics and Human Development, Michigan State University, East Lansing, MI 48824 USA

**Keywords:** Flint water crisis, Developmental patterns, Preschool, Executive functioning, Service

## Abstract

**Objective:**

Children in Flint, Michigan, have experienced myriad sociodemographic adversities exacerbated by the Flint water crisis. To help inform child-focused prevention and intervention efforts, we aimed to describe patterns of neurodevelopmental outcomes among preschoolers who experienced the Flint water crisis before age 2 years.

**Method:**

Participants were 170 preschoolers who completed a comprehensive neurodevelopmental assessment battery, including directly administered measures of cognitive and executive functioning and maternal-report of adaptive skills and behavioral problems. We used latent profile analysis to derive subgroups. Multivariate multinomial logistic regression was conducted to examine the predictors of profile memberships, including child sex and maternal/family-level factors selected from an array of measured exposures using least absolute shrinkage and selection operator regression.

**Results:**

Three latent profiles were identified: *Profile 1—*relative weakness in all domains (50%); *Profile 2—*normative functioning in all domains (34.1%); and *Profile 3—*relative strengths in executive function and behavior (15.9%). Profile 1 showed lower scores across cognitive and behavioral domains. Profile 2 demonstrated abilities within the normal range across domains. Profile 3 showed relative strength in executive functioning with few behavior problems, despite lower cognitive performance. Children across all profiles showed adaptive behavior in the adequate range. Child sex and maternal IQ were significant predictors of profile membership.

**Conclusions:**

Children in Flint demonstrated diverse patterns of development in the face of sociodemographic and environmental adversities. Comprehensive screening and neurodevelopmental profiling of children in this at-risk population are needed to identify areas of needs and inform appropriate service delivery.

**Supplementary Information:**

The online version contains supplementary material available at 10.1186/s11689-021-09377-y.

## Background

There are few places in the USA where the inequitable distribution of resources and opportunities for children are more striking than Flint, MI. Compared to a rate of 17% in the USA [1], about 70% of children in Flint live in poverty [2]. These children have historically experienced greater exposure to structural racism, malnutrition, parental unemployment, neighborhood violence, and limited access to health and educational services [3, 4]. Compounding these already significant and long-standing adversities facing the Flint community, the Flint water crisis, which began in April 2014, added unprecedented trauma including governmental indifference, population-wide lead exposure, an outbreak of deadly legionnaire’s disease, and increased stress [5, 6]. Emblematic of public health and environmental injustice, the Flint water crisis amplified socioeconomic and racial inequalities exposing this vulnerable population to a heightened risk for poor neurodevelopmental outcomes. While multiple agencies mobilized to provide support and resources to children and families in Flint [3], relatively little was known about their specific needs and areas of strength. The current study was initiated to understand variation across neurodevelopmental outcomes in this cohort of preschool-aged children and to understand predictors of those outcomes in order to inform early identification and intervention processes.

Prior studies have documented racial and/or socioeconomic inequalities in multiple exposures that matter for child developmental outcomes and are in part determined by upstream, social factors, including family poverty, parental incarceration, residential segregation, and neighborhood violence and quality [7–10]. In turn, these factors influence many aspects of the child’s environment known to impact neurodevelopment, including levels of cognitive enrichment, quality early childcare and schooling, psychosocial stress, and exposure to a variety of toxins (e.g., lead, air pollution, phthalates, and flame retardants) [11–15]. While these adverse environmental exposures further increase the risk of suboptimal developmental outcomes, children are likely to show varying degrees of susceptibility partly due to the presence or absence of protective factors (e.g., positive parenting practices, high-quality early education, adequate nutrition) [16–19]. Moreover, aspects of neurodevelopment are rarely affected in isolation, and the impact of toxic chemical and non-chemical exposures on multiple biological and psychological processes can lead to different patterns of functioning that may manifest across the life course [10, 20, 21]. Recognizing the multifactorial influences on multiple domains of early childhood neurodevelopment, it is necessary to consider the core aspects of early development simultaneously to identify subgroups of children that show similar neurodevelopmental patterns.

Latent profile analysis (LPA) is one approach for identifying such subgroups. Unlike variable-oriented approaches that often treat individual differences as nuisances, LPA is a person-centered, model-based approach to derive latent subgroups with similar patterns of performance across multiple neurodevelopmental domains within a given sample. LPA has been employed to describe variable neurodevelopmental patterns within at-risk populations, such as sensory subtypes in children with autism spectrum disorder [22] and executive functions in children with attention-deficit hyperactivity disorder (ADHD) [23]. In the current study, we used LPA to characterize neurodevelopmental patterns in a sample of preschool-aged children living in Flint, MI, and exposed to the water crisis postnatally. We included scores from standardized measures of core early developmental aspects known to be impacted by lead and other social and environmental exposures (i.e., cognitive functioning, executive functioning, adaptive behavior, and behavior problems) with the goal of identifying outcome profiles and their correlates to inform service and support allocations for this vulnerable group of children.

## Method

### Participants

Mother-child dyads were invited to participate if the child was born between 3/1/2012 and 4/24/2014 (before the water source change) and if the child resided in the City of Flint and received water from the Flint water distribution system for some time between 4/25/2014 and 10/15/2015. These dates were chosen to include those children exposed to the water crisis in the early postnatal period (within the first two years of life) who were also old enough to complete robust, direct assessments of neurodevelopment at the in-person visit.

While implementing a fully representative sample strategy was beyond the scope of this study, we took several steps to improve the representativeness of our sample. Our primary method of contacting families was through lists of eligible children provided by the Michigan Department of Health and Human Services (MDHHS) Michigan Care Improvement Registry (MCIR; including all children who ever received immunizations) and the Hurley Medical Center the only Children’s Hospital. Together, the two lists produced 2005 children making up for the majority of total population who might be eligible for the study estimated based on birth data from Michigan Department of Health & Human Services, Division of Vital Records & Health Statistics.^1^ Study invitation letters were mailed to potentially eligible dyads with an opt-out response option, and then, a series of attempts were made to contact families through telephone and email invitations. Mother-child dyads were also recruited through advertisements at local preschools, elementary schools, community centers, and parent groups, as well as through social media and word of mouth.

**Fig. 1:**
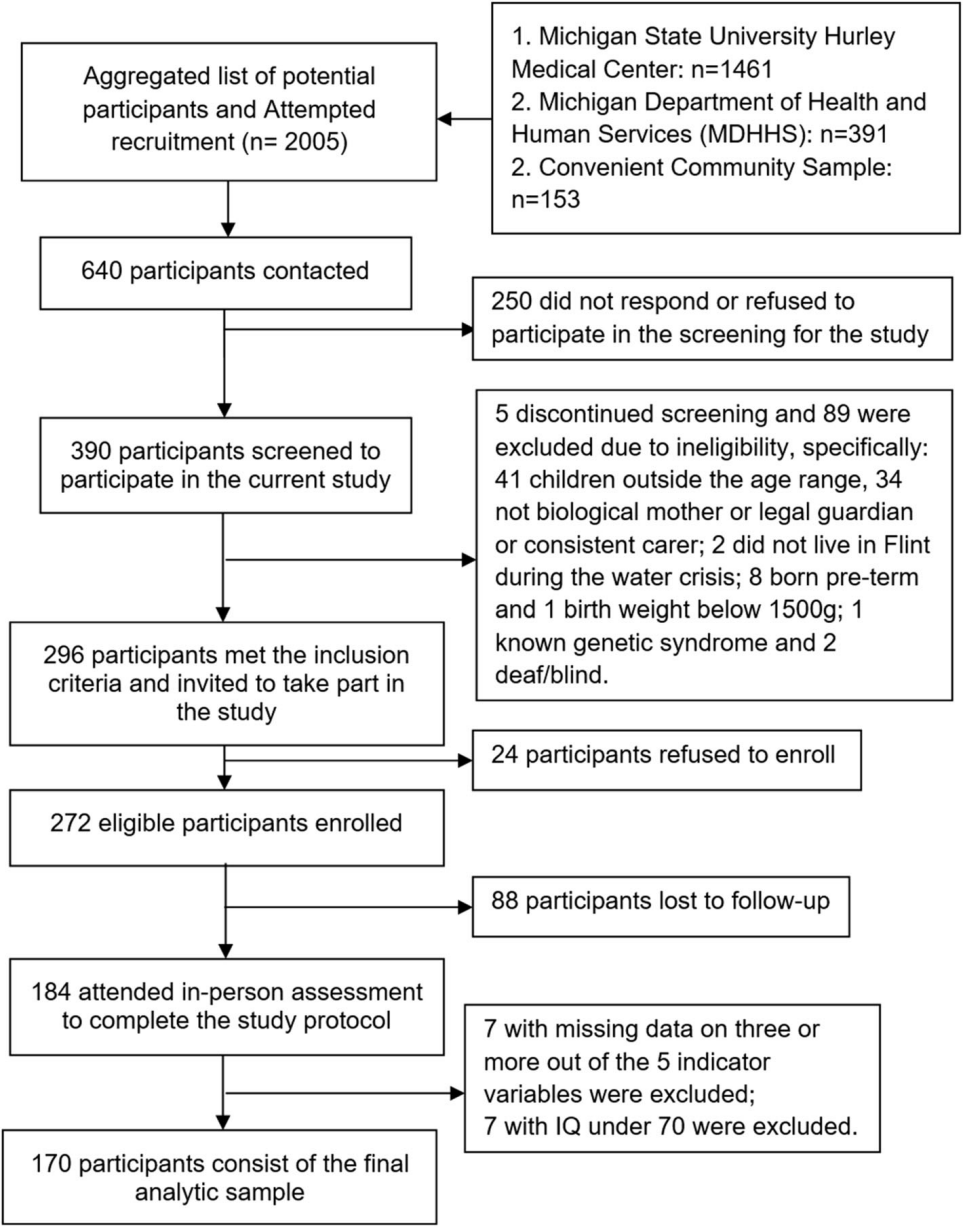
Participant recruitment and selection flowchart

Families who expressed interest in participation were screened for eligibility. Mothers were excluded if they were not the biological mother of the child, if they were non-English speaking, or if they did not reside with and/or consistently care for their child since birth, to ensure the accuracy of retrospective data collected on birth outcomes, infant feeding practices, and water use history. Children were excluded if they were wards of the state, if their birth weight was less than 1500 g, if gestational age was less than 32 weeks, or if they had a known genetic syndrome. To ensure that they could validly complete the tests included in the direct assessment battery, children were also excluded if they were currently nonverbal, or had significant hearing or visual impairments. A total of 390 mother-child dyads participated in screening, of whom 284 were determined to be eligible, and 272 agreed to enroll. Of those, 184 attended an in-person assessment. Children with measured IQs in the range of intellectual disability (IQ<70; *n*=7; see Table S1 for detailed scores on the neurodevelopmental measures) were excluded from the LPA due to concerns about the validity of their neurodevelopmental assessments given the non-clinical setting, where research assistants who were less experienced in managing challenging behaviors and eliciting responses from children with more prominent delays administered the assessments. We excluded those missing three or more of five LPA indicators prior to imputation (see Fig. 1 flowchart for details on participant recruitment and selection for analyses).

### Procedure

We developed a comprehensive assessment battery to evaluate skills across multiple developmental domains and collected parent-report information on child development and behaviors from mothers. Mothers completed online surveys, and mother-child dyads completed in-person visits with trained research assistants that included direct assessments of children and additional in-person surveys completed by the mother. All research assistants received training and supervision in the administration of direct assessments from a licensed clinical psychologist.

The institutional review board at the authors’ institutions reviewed and approved the study protocol, and the MDHHS and Hurley Medical Center approved the affiliated recruitment protocol. Verbal informed consent from mothers was obtained prior to screening and online data collection. Written informed consent and parent permission were obtained from the mother at the beginning of study visits, and verbal assent was obtained from the child.

### Measures

#### Neurodevelopmental assessment

The neurodevelopmental assessment battery was designed to capture comprehensive early developmental aspects of cognitive and language development, adaptive behaviors, and behavior problems. The following measures were included in this battery.

*Wechsler Preschool and Primary Scale of Intelligence–Fourth Edition (WPPSI–IV)* is a widely used and well-validated intelligence test designed for children ages 2 years, 6 months to 7 years, 7 months [24]. The current analysis used the norm-referenced standard scores corresponding to full-scale IQ (FSIQ) with a mean of 100 and Standard Deviation (SD) of 15. Composite scores on specific cognitive aspects, verbal comprehension, fluid reasoning, and working memory, were also generated.

*NIH Toolbox Cognitive Batteries* [25] are sets of measures designed to assess cognition in the general population with brief tasks that could be administered on tablets. Child participants were administered the two subtests from the Early Childhood Cognition Battery on executive functioning: Flanker Inhibitory Control and Attention Test (Flanker, measuring inhibitory control) and Dimensional Change Card Sort Test (DCCS, measuring cognitive flexibility).

*Child Behavior Checklist for Ages 1.5–5* (CBCL 1.5–5; [26]) is a parent-report measure on child behaviors, with 99 problem items rated from 0 “Not true” to 2 “Very true.” Raw scores and norm-referenced *T*-scores can be derived for the Internalizing, Externalizing, and Total Problems scales; syndrome scales; and DSM-oriented scales. *T*-scores of 64 and above for Internalizing, Externalizing, and Total Problems scales are considered to be in the clinical range of concern.

*Vineland Adaptive Behavior Scale* (Vineland-II; [27]; Vineland 3; [28]). Both Vineland-II and Vineland 3 were used in the current study. Initially, the Vineland-II comprehensive interview form was administered over the phone with the biological mother by trained study staff (*N*=40). Based on the feedback from the study staff and participants (e.g., scheduling difficulty, length of assessment), we switched to using the Vineland-3 online parent report form for the rest of the participants to reduce the burden. Both versions yield domain standard scores for Communication, Daily Living Skills, Socialization, and Motor skills, as well as an Adaptive Behavior Composite (ABC) score. Supplementary Table S1 includes the domain level standard scores and ABC score on both versions/administration formats, showing largely similar ranges and distributions.

*Clinical Evaluation of Language Fundamentals Preschool-Second Edition* (CELF-P-2 [29]) evaluates the language skills of preschool-aged children and provides norm-referenced standard scores for core language skills based on their performance on three subsets (e.g., sentence structure, word structure, and expressive vocabulary).

*Social Responsiveness Scale-2* (SRS-2 [30]) is a 65-item parent-report questionnaire designed as a continuous measure of “autistic traits.” The preschool form was used in the current study, and raw scores from the Social Communication Impairments and Repetitive and Restricted Behaviors domains were commonly used in analyses.

#### Demographic and socio-environmental exposures

Demographic and socio-environmental variables were collected through parent report. These included household income level, maternal demographic characteristics (i.e., educational attainment, employment status, and relationship status), maternal mental health (i.e., symptom levels of depression and stress, substance abuse, experiences of domestic violence, social support, and life orientation), maternal parenting characteristics (i.e., child-rearing practices: nurturance and conflict, knowledge of effective parenting, criticism toward their child), home environment (i.e., levels of cognitive stimulation), and any adverse childhood experiences. Maternal cognitive abilities were measured using NIH Toolbox batteries [25] and included as a candidate predictor given that previous studies showing association between maternal IQ and child outcomes [31–33]. See supplementary Table S3 for details of each measure used to assess each exposure variable. Additionally, child biological sex was also considered as a risk factor given that males are at greater risk for neurodevelopmental disorders [34] and more susceptible to environmental exposures [35–38].

#### Analysis plan

##### Latent profile indicators

We used a combination of theory and data-driven approaches to select LPA indicators from the full neurodevelopmental battery, focusing on measures that capture different domains of child neurodevelopmental functioning comprehensively. We chose to only include total scores of measures that summarized broad domains of functioning (e.g., cognitive performance, total behavior problems) rather than subscales scores (e.g., verbal IQ, internalizing behavior), because (1) subscale scores and total scores were highly correlated (see Tables S5-S8) and total scores are meant to capture the information in the subscale scores and (2) the total scores are more reliable and show higher convergent validity with other tests of the same construct (e.g., cognitive functioning, [39]; adaptive skills, [40]). We also evaluated correlation coefficients between the different total scores across measures to ensure that indicators included were somewhat distinct, which we operationalized as absolute values of correlation coefficients of less than 0.7 (See supplementary Tables S4). This process resulted in five global measures of child neurodevelopment being entered into the LPA models for classification: WPPSI FSIQ (general cognitive performance), Flanker age-corrected standard score (executive function, attention), DCCS age-corrected standard score (executive function, attention shifting), CBCL total problem T-score (general behavior problems), and Vineland ABC standard score (adaptive behavior).

##### Latent profile analyses

We used the R package *mice* (multivariate imputation by chained equation; [41]) to handle missing data with multiple imputation. With the complete imputed dataset, LPA was conducted using R package *mclust* [42]. Models with different numbers of profiles (1-profile to 9-profile models) and different parameterization of covariance matrices were fitted. The fit indicators (Bayesian Information Criterions, BICs) of models were examined to select the best model (i.e., with the highest BIC). Once the best model was identified, children were assigned to their most likely profiles based on the posterior probabilities. We describe the differences in the five LPA indicators across the resulting profiles and compare profiles on additional developmental and behavioral measures in the battery using ANOVA with *post hoc* tests. To adjust for multiple comparisons, we applied a Bonferroni corrected significance threshold of 0.0024 for 21 comparisons.

##### Identifying predictors of profile membership

To select salient predictors among the array of socio-environmental factors described above, we applied least absolute shrinkage and selection operator (LASSO) regression with categorical profile membership as the dependent variables. For the LASSO regression, all continuous variables were standardized with a mean of 0 and SD of 1 to be so that variables were on the same scale for the LASSO model estimation, and the categorical variables were dummy coded. Multivariate multinomial logistic regression models were fit to examine the association between the LASSO-selected predictors and profile membership; child sex was added to the models given that it is an important predictor of neurodevelopmental outcomes and child age and maternal age were added as covariates. We conceptualized race as a social construct confounded with many other socio-demographic variables we already included in the LASSO regression for predicting profile membership. Given that one cannot intervene on race but rather the many disproportionate burdens with which it is associated, we did not include race as a primary predictor. However, we did conduct sensitivity analysis with race (non-African American vs. African American) included in the multinomial logistic regression.

## Results

Of the 177 children who scored higher than 70 on the WPPSI FSIQ, seven were excluded due to missing data for more than three out of the five classifying variables listed (more than 60% missing). This resulted in a final analytic sample of *N*=170. Mean child age at the time of assessment was 5.47 years (SD=0.38) (Table 1). About half (51.18%) of the children were male, and a majority (67.93%) were African American, with a large proportion of children living in low SES households (52.18% with annual income below $15,000; 47.64% of the mothers with a high school degree or less; 76.63% of mothers were not partnered). Demographics of our sample largely reflected those of the total population of families in Flint, with our sample having a slight over-representation of African Americans (see Supplement Table S9).

**Table 1:** Demographic information and descriptive characteristics of the analytic sample (*N*=170)

		***N***	**%**
**Child gender**	Male	87	51.18
**Child race**	White	26	15.29
	Black or African American	117	68.82
	Multi-race	23	13.53
	Other	2	1.18
	Missing	2	1.18
**Child ethnicity**	Hispanic	14	8.24
**Maternal education**	Less than high school	21	12.35
	High school or GED	60	35.29
	Vocational/technical school or some college/associate degree	72	42.35
	College or graduate degree	15	8.82
	Missing	2	1.18
**Maternal employment**	Working full-time	29	17.06
	Working part-time	58	34.12
	Not employed	60	35.29
	Disabled, not able to work	19	11.18
	Retired	1	0.59
	Missing	3	1.76
**Maternal marital status**	Single moms	133	78.24
	Missing	3	1.76
**Household income**	Less than $10,000	59	34.71
	$10,000–$15,000	31	18.24
	$15,001–$25,000	19	11.18
	$25,001–$35,000	16	9.41
	$35,001–$45,000	8	4.71
	$45,001–$55,000	8	4.71
	$55,001 and up	9	5.29
	Don’t know	14	8.24
	Missing	6	3.53
		**Mean (SD)**	**Range**
**Child age (*N*=170)**		5.47 (0.38)	[4.05, 6.04]
**Mother age (*N*=170)**		31.39 (6.38)	[21.25, 52.95]
**WPPSI FSIQ Standard(Std) Score (*N*=167)**		88.99 (11.89)	[70, 120]
**DCCS Std Score (*N*=162)**		94.43 (13.03)	[59, 117]
**Flanker Std Score (*N*=158)**		94.72 (13.36)	[62, 135]
**CBCL Total Problems T Score (*N*=164)**		51.79 (14.67)	[28, 93]
**Vineland ABC score (*N*=146)**		95.66 (15.21)	[51, 140]

*Vineland ABC score*, Vineland adaptive behavior composite standard score

### Person-center LPA results

Of the 1-profile to 9-profile models with 14 different covariance parameterizations tested, the 3-profile model with a diagonally distributed covariance structure was selected as the best model using BIC criteria (see Supplementary Table S10). In the 3-profile model solution, half of the sample was classified to *Profile 1* “Relative weakness in all domains” (50%), about a third of the sample to *Profile 2 “*Normative functioning in all domains” (34.1%), and the smallest proportion to *Profile 3* “Relative strengths in executive function and behavior” (15.9%).

**Fig. 2:**
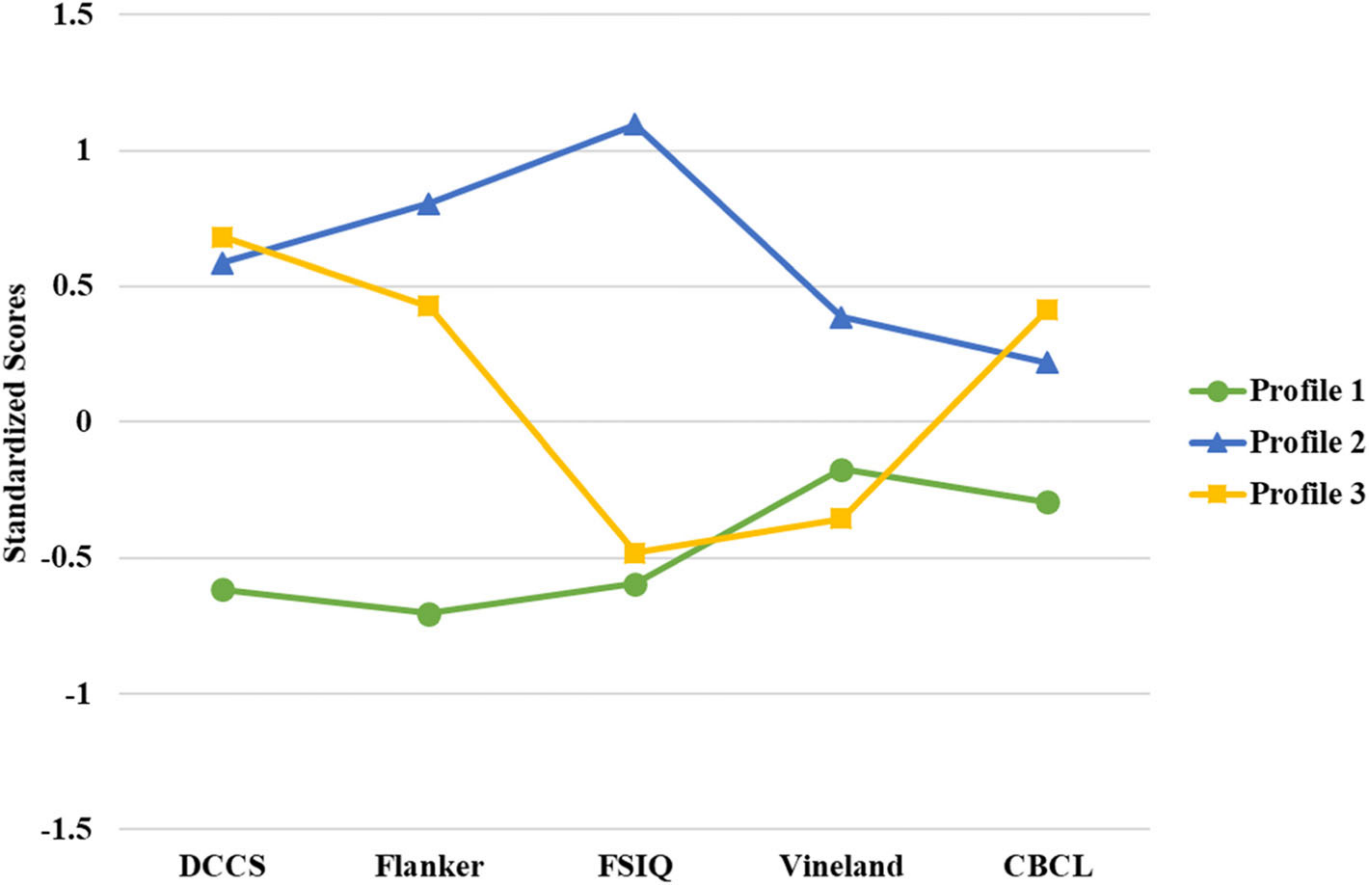
Line plot with standardized scores of the three profiles across the five indicator variables included in the LPA. Note: The figure shows the standardized scores of five indicator variables in the model by profiles: NIH Toolbox Dimensional Change Card Sort Test (DCCS) and Flanker Inhibitory Control and Attention Test (Flanker), WPPSI full scale IQ (FSIQ), Vineland Adaptive Behavior Composite Score, and reversed CBCL total problems scores (the scores were reversed to be in the consistent direction with other measures on the graph, with higher scores indicating better performance/fewer problems)

The three profiles showed statistically significant differences across the five indicator variables, with the largest differences in cognitive and language abilities (Fig. 2, Table 2). Notably, all three profiles were characterized by adaptive behavior skills in the “Adequate” range as measured by the Vineland ABC score. Further, the three profiles were compared across all available measures of neurodevelopmental domains to provide more detailed depictions of the profiles: Profile 1 “Relative weakness in all domains” showed the lowest scores on measures of cognitive performance and the highest scores on the CBCL total problems with adaptive behavior skills in the adequate range (Table 2). Additionally, Profile 1 showed the lowest language skills on the CELF-P-2 and significantly higher social communication impairments on the SRS compared to Profile 2. Profile 1 also has the highest percentage of children meeting the clinical cut-offs on CBCL problem scales (32.84% on externalizing problems and 38.24% on internalizing problems); Profile 2 “Normative functioning in all domains” showed the highest cognitive performance (WPPSI FSIQ mean 102.02, SD 7.20, and 100% above the score of 85) and significantly higher executive functioning and adaptive functioning than Profile 1. On assessments not included in the LPA, children in Profile 2 scored the highest in the language skills on the CELF-P-2 (Table 2); Profile 3 “Relative strengths in executive function and behavior” had a unique profile across the five LPA indicator variables. Children classified in Profile 3 had lower scores on the WPPSI, similar to Profile 1. However, they had high scores on executive functioning measures (the NIH Toolbox Flanker and DCCS) that were, on average, comparable to or even higher than Profile 2 (see Table 2). Moreover, in the behavioral domains, Profile 3 had the lowest severity on CBCL behavioral problem scores and SRS social impairment scores, with the fewest cases meeting the clinical cut-off for internalizing (8.33%), externalizing (0%), or total problems (0%) on the CBCL.

**Table 2:** Descriptive statistics on child measures across profiles

Variable	Profile 1 (*N*=85; 50%)	Profile 2 (*N*=58; 34.12%)	Profile 3 (*N*=27; 15.88%)	p*	Effect size (*η*^2^)	Post hoc comparisons*
	Mean (SD), IQR	Mean (SD), IQR	Mean (SD), IQR			
**Child age**	5.42 (0.4)	5.48 (0.35)	5.6 (0.35)	0.0785	0.03	
**Child sex (% of male)**	56.47%	37.93%	62.96%	0.0383		
**WPPSI FSIQ Std Score**	81.9 (7.96), 14	102.02 (7.2), 9	83.26 (4.9), 7	<.001*	0.63	2>1; 2>3
**% of FSIQ≥85**	36.14%	100%	33.33%			
**DCCS Std Score**	86.41 (12.03), 20	102.07 (9.35), 9	103.32 (4.44), 9	<.001*	0.38	2>1; 3>1
**Flanker Std Score**	85.31 (11.14), 25	105.47 (8.59), 12	100.44 (3.98), 0	<.001*	0.50	2>1; 3>1
**CBCL Total Problems Tscore**	56.1 (15.9), 26	48.57 (13.27), 16	45.69 (8.99), 16	0.0007*	0.09	1>3
**% met clinical cut-off**	36.25%	10.34%	0%			
**Vineland ABC score**	93.03 (16.62), 17	101.56 (13.15), 17.5	90.24 (9.79), 11	0.0014*	0.09	2>1
**WPPSI GA Std Score**	81.55 (9.54), 14	100.58 (8.31), 11	82.74 (5.91), 8	<.001*	0.52	2>1; 2>3
**WPPSI NVI Std Score**	81.89 (10.15), 11	99.68 (10.45), 15	83.84 (6.58), 9	<.001*	0.42	2>1; 2>3
**WPPSI VCI Std Score**	84.46 (10.9), 14	102.6 (11.28), 18	86.56 (8.01), 14	<.001*	0.39	2>1; 2>3
**WPPSI FRI Std Score**	82.33 (13.61), 19	96.63 (13.85), 24	84 (11.12), 7	<.001*	0.20	2>1; 2>3
**WPPSI WM Std Score**	87.71 (12.01), 15	101.63 (11.78), 20	95.31 (11.8), 16	<.001*	0.22	2>1
**CELF-P-2 Core Language Std Score**	85.02 (11.89), 13	103.1(10.5), 16	90.16(8.56), 8	<.001*	0.36	2>1; 2>3
**Vineland Motor Std Score**	90.96 (16.86), 19	100.54 (14.38), 17.5	88.67 (15.66), 24	0.0013*	0.09	2>1
**Vineland Communication Std Score**	90.52 (17.05), 21	98.42 (11.62), 12	91.67 (10.89), 14	0.0101	0.06	
**Vineland Daily Living Std Score**	97.05 (17.87), 25	105.58 (13.53), 15	93.71 (10.46), 10	0.0021	0.08	
**Vineland Socialization**	95.74 (16.71), 17	101.66 (15.91), 25	92.67 (12.04), 20	0.0416	0.04	
**Std Score**						
**SRS-2 Repetitive Behavior Raw Score**	7.91 (6.8), 8	5.02 (4.72), 5.5	4.31 (2.66), 3	0.0026	0.07	
**SRS-2 Social Communication Impairment Raw Score**	46.37 (18.76), 29	34.59 (18.44), 25.5	37.62 (13.58), 16	0.0008*	0.09	1>2
**CBCL Externalizing Raw Score**	17.31 (11.2), 16	10.82 (9.1), 13	9.4 (6.57), 10	0.0002*	0.11	1>2; 1>3
**% of met clinical cut-off**	32.84%	10.90%	0%			
**CBCL Internalizing Raw Score**	14.22 (12.2), 17	9.19 (9.02), 6	7 (5.28), 4.5	0.003	0.08	
**% of met clinical cut-off**	38.24%	12.96%	8.33%			

*FRI* fluid reasoning, *FSIQ* full scale IQ, *GA* general ability, *IQR* interquartile range, *NVI* nonverbal, *SD* standard deviation, *SRS* Social Responsiveness Scale-2, *VCI* verbal comprehension, *WM* Working Memory

^*^Bonferroni corrected threshold *p*<0.0024

### Predictors of profiles

We selected Profile 2, the normative functioning group, to serve as the reference group for both the LASSO regression and multinomial logistic regression model to examine risk factors associated with subgroup membership. Of the predictors examined, the LASSO model selected two predictors of profile membership: maternal IQ and household income level based on the model fit index of AICC corrected for small sample bias (AICC=135.51, BIC=143.68). The multivariate multinomial logistic regression model included child sex, maternal IQ, and household income while controlling for child age and maternal age (*R*^2^=0.25). Compared to the normative functioning Profile 2, girls were less likely to be in Profile 3 “Relative strength in executive function and behavior” (OR=0.26, *p*=0.03), and children of mothers with lower IQ were more likely to be in Profile 1 “Relative weakness in all domains” (odds ratio [OR]=0.46, *p*=0.004) (see Table 3). Children from higher-income households were more likely to be in Profile 2 (normative functioning) than in Profile 1 “Relative weakness in all domains” (see Table 3), though findings were marginally significant (OR=0.55, *p*=0.058). In sensitivity analysis, race was not a significant correlate of profile membership nor did it significantly contribute to variance explained (△*R*^2^=0.03; ps>0.05).

**Table 3:** Results from multinomial logistic regression with Profile 2 as the reference group

			Profile 1 “Relative weakness in all domains”			Profile 3 “Relative strengths in executive function and behavior”		
			Odds ratio	95% CL	*p*	Odds ratio	95% CL	*p*
LASSO-selected predictors	Maternal IQ		0.46	[0.27, 0.78]	0.004	0.85	[0.44, 1.63]	0.626
	Household income		0.55	[0.30, 1.02]	0.058	0.65	[0.32, 1.33]	0.240
Demographic predictors	Child sex	Male	[Reference]	--	--	[Reference]	--	--
		Female	0.43	[0.18, 1.03]	0.059	0.26	[0.08, 0.89]	0.032
Covariates	Child age		0.81	[0.51, 1.29]	0.383	2.34	[1.01, 5.43]	0.045
	Maternal age		1.33	[0.86, 2.05]	0.196	0.97	[0.53, 1.79]	0.923

## Discussion

We aimed to understand variability neurodevelopmental profiles within a well-characterized sample of preschool children in Flint, MI. While some children showed significant deviations from same-aged peers in one or more developmental domains assessed, others showed profiles suggesting limited neurodevelopmental risk. LPA revealed three profiles characterized by varying levels of functioning across developmental domains (cognitive abilities, executive functioning, behavior problems, and adaptive behavior skills): Profile 1 “Relative weakness in all domains,” Profile 2 “Normative functioning in all domains,” and Profile 3 “Relative strengths in executive function and behavior”.

Children in Profile 1, representing half of the sample, showed the lowest scores in all domains of cognitive abilities, language abilities, and executive functioning, as well as elevated behavior problems (i.e., greater social impairment and externalizing problems). Nonetheless, children in Profile 1 showed adequate levels of adaptive behaviors on average, indicating that their everyday functioning is relatively intact even in the context of other delays and difficulties. Profile 3 had similarly low cognitive and language abilities as Profile 1. However, they exhibited the highest executive functioning (within the average range) and fewest behavior problems (with none meeting clinical cut-offs for either total or externalizing problems). This is consistent with previous findings showing that better executive function is associated with higher emotion regulation skills [43] and fewer behavior problems [44]. Given the observed delays in at least some developmental domains for children in Profiles 1 and 3, early screening and surveillance efforts are warranted to identify and support children who might not otherwise come to the attention of professionals. Especially for children like those in Profile 3 with few parent-reported behavior problems (i.e., CBCL), their parents might be less likely to seek service [45, 46], and thus, these children might be less likely to receive early intervention. Children in Profile 2, the normative functioning class, exhibited average-range abilities across the multiple neurodevelopmental domains measured in the current study. These children demonstrate resilience in the face of multiple early adverse experiences. Many previous LPA studies with young children at risk (e.g., children born preterm [47, 48] or attending special education services [49]) identified three to four profiles, with the largest group performing within the average range (e.g., often named as the normative group or typically developing group). However, in the current sample, the normative functioning Profile 2 takes up only around one-third of the sample, whereas the lowest-performing Profile 1 was the largest group. These findings possibly indicate that more children in the current Flint sample experience delays across neurodevelopmental domains as a result of the water crisis and many other socio-environmental exposures [2, 3].

These different outcome profiles identified by the LPA also highlight the need for additional research to understand differential susceptibility to multiple adversities and potential sources of resilience. In our exploratory investigation of possible predictors of profile memberships, we identified child sex and maternal IQ as significant predictors, and household income level as a marginally significant predictor. Consistent with previous research showing a male-dominant sex ratio for neurodevelopmental disorders [34, 50], Profile 3, with delays in cognitive and adaptive functioning, had a higher proportion of males compared to the normative Profile 2. We also found that children whose mothers had lower cognitive functioning were more likely to be in Profile 1 than in the normative Profile 2. These findings are consistent with previous studies showing positive associations between maternal IQ and child outcomes [33, 51, 52]. Taken together with the marginally significant association with household income, children in Profile 1 would likely benefit from a more enriched early learning environment with comprehensive family-level support to empower mothers who themselves might face increased challenges related to lower cognitive functioning and/or living in poverty.

Our study has several limitations. With only one data point, the current study provides a snapshot of the neurodevelopmental profiles of a sample of Flint children who were born before the onset of the water crisis. Not surprisingly, given our study population’s life circumstances, we had difficulties reaching and recruiting families to participate in the current study (only 184 mother-child dyads completed the full study protocol out of 640 contacted), raising concerns about potential sampling bias. It is possible that parents who were already concerned about their children’s development were more likely to participate so that they could receive a comprehensive neurodevelopmental assessment, and/or it is possible that families facing the greatest hardships (and whose children may also have been at increased risk for neurodevelopmental difficulties) were less likely to participate because of the burden associated with the study protocol. Further, our findings might not be representative of children who experience additional health challenges (e.g., born preterm or with low birth weight, nonverbal, deaf or blind) as we excluded those children due to concerns about the validity of neurodevelopmental assessments. While subgrouping methods like LPA have well-acknowledged limitations [53, 54] and our findings might be subject to sampling bias, our final sample did, in fact, reflect the socio-demographic characteristics of the broader Flint population (see Table S9).

Findings of differential outcome profiles in the Flint sample have possible implications for understanding the development of children in other communities in USA who experience comparable socio-environmental exposures [55, 56], though replications of the profiles and profile predictors in other samples are needed. Moreover, the different neurodevelopmental profiles provide further support for multifinality in child development and suggest careful considerations of tailored interventions to meet the needs of individual children and families. Further, it is important to acknowledge that all children in Flint, regardless of current abilities, may require additional long-term supports and services given exposure to multiple psychosocial stressors (including the water crisis) and the time-lag in the manifestation of potential consequences (e.g., specific learning disabilities that may not appear until later in school age). Future opportunities for research include sequential longitudinal neurodevelopmental assessments, correlation of profiles with historic lead exposure, and evaluation of the impact of mitigating interventions and services.

## Conclusion

Collectively, our findings demonstrate the importance of considering multiple aspects of development simultaneously and the utility of person-centered approaches for evaluating the needs of children in at-risk communities. We identified three neurodevelopmental profiles among a sample of preschool-aged children who experienced the Flint water crisis, suggesting diverse developmental patterns in response to myriad social and environmental exposures, with adaptive behavior skills as a relative strength despite highly varying degrees of functioning in other domains. Therefore, comprehensive screening and neurodevelopmental profiling of children in the Flint community are needed to support appropriate service delivery. Our findings also suggest that boys as well as children with mothers with lower cognitive functioning and/or lower-levels of household income might be more vulnerable to delays in different developmental domains. Future longitudinal studies are also necessary to examine the effect of the multiple environmental and chemical exposures on neurodevelopment and long-term outcome among children in Flint and other at-risk communities.

## Supplementary Information

**Additional file 1.** Supplementary tables.

## Data Availability

The datasets analyzed in the current study available from the senior author (KL) on reasonable request with permission from the granting agency and recruitment registry.

## Abbreviations

ABC: Adaptive Behavior Composite
ADHD: Attention-deficit hyperactivity disorder
AICC: Akaike Information Criterion corrected for small sample
BIC: Bayesian Information Criterions
CBCL 1.5–5: Child Behavior Checklist for Ages 1.5–5
CELF-P-2: Clinical Evaluation of Language Fundamentals Preschool-Second Edition
DCCS: Dimensional Change Card Sort Test
FSIQ: Full-scale Intelligence Quotient
MCIR: Michigan Care Improvement Registry
MDHHS: Michigan Department of Health and Human Services
LASSO: Least Absolute Shrinkage and Selection Operator
LPA: Latent profile analysis
OR: Odds Ratio
SD: Standard Deviation
SES: Socioeconomic status
SRS-2: Social Responsiveness Scale-2
WPPSI–IV: Wechsler Preschool and Primary Scale of Intelligence–Fourth Edition

## Footnotes

1 Based on geocoded Michigan birth certificate record data available through the Michigan Department of Health & Human Services, Division of Vital Records & Health Statistics, we estimated that there were about 130 live births per month in the City of Flint between 2012 and 2014. Based on our inclusion criteria, the earliest month of birth is July 2012 to be eligible, resulting in an estimated eligible population of 2860 based on 22 months of eligible births. Therefore, the total numbers on the two registry lists we recruited from would account for 70% (2005 out of 2860) of the estimated eligible population, allowing us to capture the majority of the population.
