# Supplementary material for: Neurodevelopmental profiles of preschool-age children in Flint, Michigan: a latent profile analysis
Source: J Neurodev Disord. 2021 Aug 19;13:29. doi: 10.1186/s11689-021-09377-y (PMC8377967; doi:10.1186/s11689-021-09377-y)
Supplement: Supplementary file 1 — Additional file 1. Supplementary tables. [file 11689_2021_9377_MOESM1_ESM.docx]

**Supplementary Material**

Table S1

*Descriptive Statistics of Scores across Measures of Children with Full-Scale IQ under 70*

| Variable | N | Mean | Std Dev | Minimum | Maximum |
| --- | --- | --- | --- | --- | --- |
| WPPSI FSIQ Std Score | 7 | 66.29 | 2.69 | 62 | 69 |
| Vineland ABC score | 7 | 90.14 | 11.26 | 75 | 108 |
| CBCL Total Problems Tscore | 6 | 43.83 | 7.49 | 30 | 52 |
| DCCS Std Score | 5 | 84.00 | 8.94 | 78 | 98 |
| Flanker Std Score | 7 | 79.29 | 9.57 | 62 | 87 |
| CELF-P-2 Core Language Std Score | 7 | 77.00 | 4.32 | 71 | 83 |
| SRS-2 Repetitive Behavior | 6 | 5.17 | 4.62 | 1 | 11 |
| SRS-2 Social Communication | 6 | 48.67 | 15.53 | 29 | 70 |
| CBCL Externalizing Raw Score | 6 | 7.00 | 3.16 | 2 | 11 |
| CBCL Internalizing Raw Score | 5 | 8.20 | 7.73 | 0 | 21 |

Table S2

*Descriptive Statistics on Both Versions of Vineland Adaptive Behavior Scale in the Current Sample*

| **Vineland Version** | **Domains** | **N** | **Mean** | **Std Dev** | **Range** |
| --- | --- | --- | --- | --- | --- |
| Vineland-2  Comprehensive Interview | Motor | 40 | 91.3 | 14.95 | [49,121] |
|  | Communication | 40 | 94.7 | 13.77 | [61,134] |
|  | Daily Living Skills | 40 | 95.75 | 16.00 | [48,134] |
|  | Socialization | 40 | 97.33 | 15.14 | [53,130] |
|  | Adaptive Behavior Composite | 40 | 93.75 | 13.65 | [51,133] |
| Vineland-3 | Motor | 116 | 93.94 | 16.99 | [48,128] |
| Parent Report Survey | Communication | 118 | 91.43 | 16.39 | [36,140] |
|  | Daily Living Skills | 118 | 99.81 | 16.61 | [50,140] |
|  | Socialization | 118 | 96.45 | 17.77 | [42,135] |
|  | Adaptive Behavior Composite | 117 | 94.98 | 16.42 | [48,140] |

Note: Std Dev=Standard Deviation

Table S3

| Variables | Measures |
| --- | --- |
| Maternal educational level | High school graduate and below vs. some college and above |
| Maternal employment status | Working (including full-time and part-time) vs. not working (including unemployed and retired) |
| Maternal relationship status | Single vs. partnered/in a relationship |
| Household income level | OECD income levels were used by dividing annual income level by the household sizes (determined by assigning a value of 1 to the household head, of 0.5 to each additional adult member and of 0.3 to each child) (Hagenaars et al., 1994; OECD, n.d.). |
| Maternal mental health: depression and stress | Depression was measured on the Center for Epidemiological Studies-Depression Scale (CES-D; Radloff, 1977)); Stress was measured on the Perceived Stress Scale (PSS; Cohen et al., 1983) |
| Number and satisfaction of social support received by mothers | Social Support Questionnaire (SSQ; Sarason et al., 1983) is used to measure the respondent’s perceived level of social support and to measure their satisfaction with this support. |
| Maternal substance abuse | CAGE Adapted to Include Drugs (CAGE-AID; Brown and Rounds, 1995) is used to screen the respondent for drug abuse. |
| Maternal experiences of domestic violence | HARK (Sohal et al., 2007) includes 4 items used to identify victims of intimate partner violence. |
| Maternal life orientation: optimism | Life Orientation Test-Revised (LOT-R; Scheier et al., 1994) is used to assess the dispositional optimism (the general expectancy of positive outcomes) and psychological resilience of the respondent. |
| Maternal child-rearing practices: nurturance and conflict | Child Rearing Practices Report (CRPR): nurturance and conflict subscales (Rickel and Biasatti, 1982) evaluate the goals, values, and attitudes of parents in regards to raising a child. |
| Maternal knowledge of effective parenting | Knowledge of Effective Parenting Scale (KEPS; Winter et al., 2012) is used to measure the respondent’s knowledge of effective parenting strategies for parents of children aged 2–10. |
| Maternal criticism toward their child | Network of Relationship Inventory (NRI) – Criticism Scale (revised parent version) (Furman and Buhrmester, 1985) is used to evaluate the frequency of criticism and harsh  feedback that the respondent gives to their child. |
| Levels of cognitive stimulation in home environment | StimQ-Parent (StimQ-P) Read Scale and Parent Verbal Responsivity Scale (Mendelson et al., 2016) evaluate cognitive stimulation in the home environment for children aged 36–60 months. |
| Adverse childhood experiences | National Survey of Children’s Health ACE questions (Bethell et al., 2017) |

Table S4

*Bivariate Correlations across Child Measures*

|  |  | 1 | 2 | 3 | 4 | 5 | 6 | 7 |
| --- | --- | --- | --- | --- | --- | --- | --- | --- |
| 1 | WPPSI FSIQ Std Score | 1 |  |  |  |  |  |  |
| 2 | DCCS Std Score | 0.34 | 1 |  |  |  |  |  |
| 3 | Flanker Std Score | 0.56 | 0.40 | 1 |  |  |  |  |
| 4 | CBCL Total Problems Tscore | -0.11 | -0.19 | -0.19 | 1 |  |  |  |
| 5 | Vineland ABC score | 0.27 | 0.03 | 0.12 | -0.28 | 1 |  |  |
| 6 | CELF-P-2 Core Language Std Score | ***0.71*** | 0.34 | 0.45 | -0.02 | 0.16 | 1 |  |
| 7 | SRS-2 Total Score | -0.27 | -0.26 | -0.20 | ***0.75*** | -0.27 | 0.25 | 1 |

Note: Std Score=Standard Score

Table S5

*Bivariate Correlations among WPPSI Scores*

|  |  | 1 | 2 | 3 | 4 | 5 | 6 |
| --- | --- | --- | --- | --- | --- | --- | --- |
| 1 | FSIQ Std Score | 1 |  |  |  |  |  |
| 2 | General Abilities Composite | ***0.93*** | 1 |  |  |  |  |
| 3 | Nonverbal Composite | ***0.87*** | ***0.74*** | 1 |  |  |  |
| 4 | Fluid Reasoning Composite | ***0.65*** | ***0.65*** | ***0.85*** | 1 |  |  |
| 5 | Verbal Comprehension Composite | ***0.82*** | ***0.89*** | 0.53 | 0.44 | 1 |  |
| 6 | Working Memory Composite | ***0.67*** | 0.47 | ***0.70*** | 0.43 | 0.40 | 1 |

Note: Std Score=Standard Score

Table S6

*Bivariate Correlations among Vineland Scores*

|  |  | 1 | 2 | 3 | 4 | 5 |
| --- | --- | --- | --- | --- | --- | --- |
| 1 | Adaptive Behavior Composite score | 1 |  |  |  |  |
| 2 | Motor Std Score | ***0.61*** | 1 |  |  |  |
| 3 | Communication Std Score | ***0.79*** | 0.45 | 1 |  |  |
| 4 | Socialization Std Score | ***0.87*** | 0.50 | 0.54 | 1 |  |
| 5 | Daily Living Std Score | ***0.93*** | 0.57 | ***0.65*** | ***0.76*** | 1 |

Note: Std Score=Standard Score

Table S7

*Bivariate Correlations among CBCL Scores*

|  |  | 1 | 2 | 3 |
| --- | --- | --- | --- | --- |
| 1 | CBCL Total Problems T-score | 1 |  |  |
| 2 | CBCL Externalizing Raw Score | ***0.92*** | 1 |  |
| 3 | CBCL Internalizing Raw Score | ***0.89*** | ***0.75*** | 1 |

Note: Std Score=Standard Score

Table S8

*Bivariate Correlations among SRS-2 Scores*

|  |  | 1 | 2 | 3 |
| --- | --- | --- | --- | --- |
| 1 | SRS total score | 1 |  |  |
| 2 | Repetitive Restricted Behaviors score | ***0.84*** | 1 |  |
| 3 | Social Communication Impairments score | ***0.99*** | ***0.75*** | 1 |

Table S9

*Demographic Characteristics of General Population in Flint, MI, 2018 based on U.S. Census*

|  |  | **2018 Flint Census Data** |
| --- | --- | --- |
| **Child Gender** | Male | 48.7%^a^ |
| **Child Race** | White | 32.08%^a^ |
|  | Black or African American | 53.58% |
|  | Other | 14.34% |
| **Child Ethnicity** | Hispanic | 5.17%^a^ |
| **Maternal Education** | Less than high school | 13.7%^b^ |
|  | High school or GED | 32.3% |
|  | Vocational/Technical School or Some College/Associate Degree | 40.2% |
|  | College or Graduate Degree | 13.8% |

Note:

1. Percentages based on children from age 0 to 5 years in Flint;
2. Percentages based on females age 25 years and above in Flint

Table S10

*BICs for the Best Three Models*

|  | VEI model with 3 profiles | VEI model with 2 profiles | EEE model with 2 profiles |
| --- | --- | --- | --- |
| BIC | -6824.47 | -6834.02 | -6835.11 |
| BIC difference |  | -9.55 | -10.64 |

Note: “VEI” models with geometric characteristics of covariance with diagonal distribution, varying volumes, and equal shape; “EEI” models with diagonal distribution, equal volume and shape (Scrucca et al., 2016).
